# A Case of Multiple Phleboliths on the Medial Side of the Right Mandible

**DOI:** 10.1155/2020/6694402

**Published:** 2020-12-27

**Authors:** Shinichi Sato, Masato Takahashi, Tetsu Takahashi

**Affiliations:** ^1^Oral and Maxillofacial Surgery, Japan Community Health Care Organization Sendai Hospital, 3-16-1 Tsutsumimachi, Aoba-ku, Sendai 981-8501, Japan; ^2^Division of Oral and Maxillofacial Surgery, Tohoku University Graduate School of Dentistry, 4-1, Seiryo-machi, Aoba-ku, Sendai 980-8575, Japan

## Abstract

A 66-year-old female patient was admitted to the orthopedic department due to a left femoral neck fracture. She received perioperative oral management prior to femoral head replacement. Laboratory blood tests indicated an elevated D-dimer level, which suggested the presence of a venous malformation. Computed tomography, magnetic resonance imaging, and short TI inversion recovery indicated the presence of multiple phleboliths medial to the right mandibular ramus. No swelling, redness, or salivary colic pain was observed. Owing to the absence of clinical symptoms, the patient elected to undergo observation of the lesion, as opposed to surgical treatment.

## 1. Introduction

Calcifications in the soft tissues of the maxillofacial region may present as sialoliths, phleboliths, calcified epitheliomas, or lymph node calcifications. Phleboliths are found in venous malformations and are thought to be caused by the calcification of intravenous thrombi [1, 2]. In this report, we describe a case in which numerous phleboliths were diagnosed in relation to the right mandible.

## 2. Case Presentation

A 66-year-old woman suffered a fall at home in January 2020 and was admitted to our orthopedic department due to a left femoral neck fracture. She was subsequently referred to our oral surgery department for perioperative oral management prior to femoral head replacement surgery. The patient provided written informed consent prior to treatment. The appropriate institutional review board waived the requirement for ethical approval.

The patient's height and weight were 160 cm and 62.9 kg, respectively, and the only medical history was the removal of a colorectal polyp.

**Figure 1:**
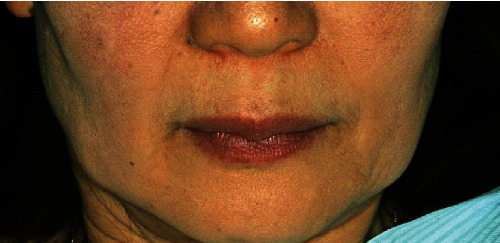
Extraoral photograph showing symmetrical facial features.

**Figure 2:**
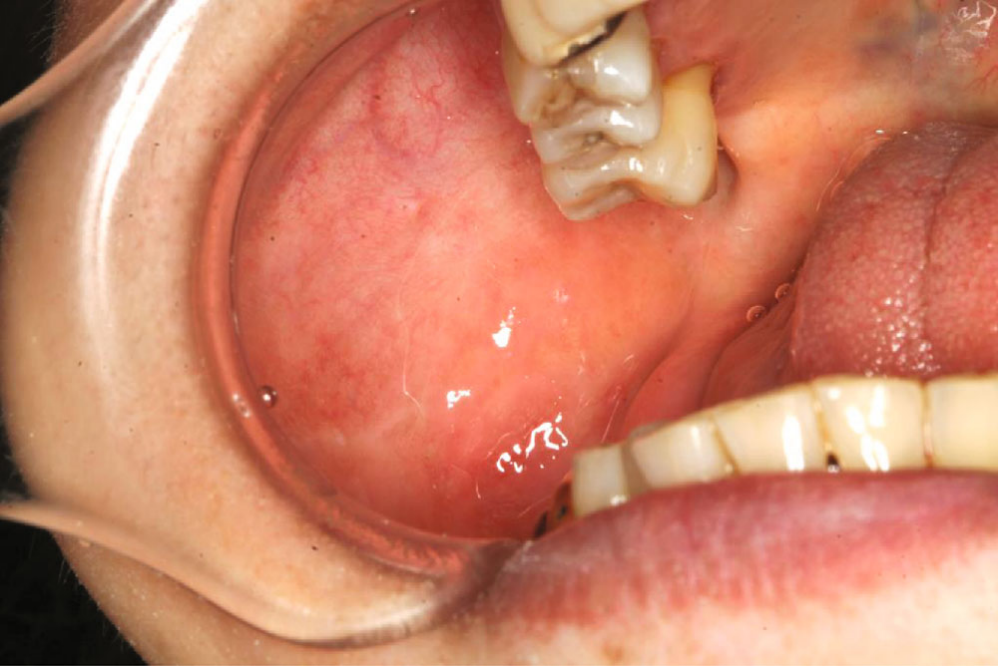
Intraoral photograph showing the absence of swelling or redness.

No facial asymmetry, swelling, or redness was observed extraorally (Figure 1). Upon intraoral examination, no redness or swelling was detected in the buccal mucosa (Figure 2). Laboratory blood tests indicated that the platelet count (31.4 × 10^4^/*μ*L) and fibrinogen level (228 mg/dL) were both within normal ranges; however, the D-dimer level (36.62 *μ*g/mL) was elevated.

**Figure 3:**
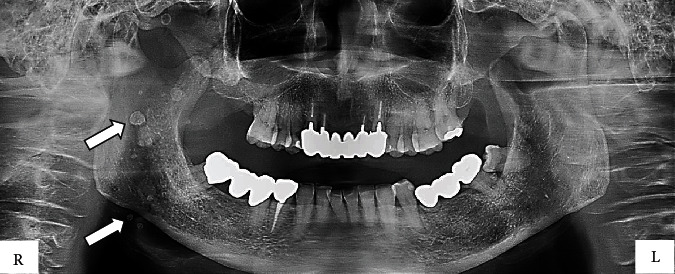
A panoramic radiograph during initial examination. Scattered opaque material suspected to be venous stones is noted. A circular opacity is evident (arrow).

**Figure 4:**
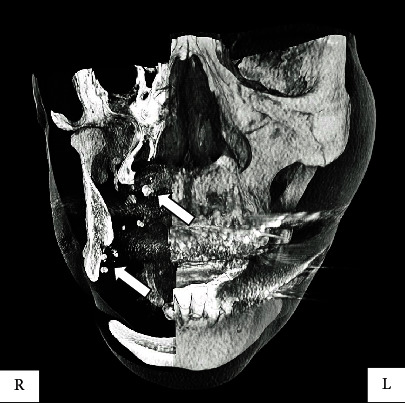
Computed tomography image showing numerous calcifications of unequal size in the medial portion of the right mandible. Circular hard tissue is evident (arrow).

**Figure 5:**
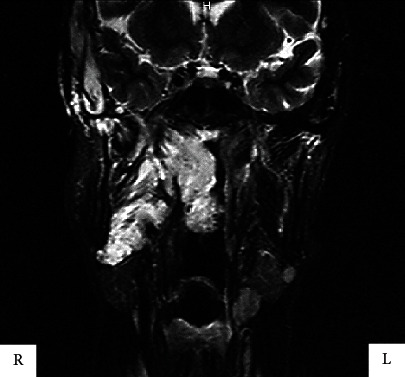
Short TI inversion recovery image showing a higher signal in the area of the lesions compared to the surrounding tissues.

Panoramic radiographs showed a dissemination of calcifications in the head and neck region that were suspected to be venous stones (Figure 3). On computed tomography, numerous calcifications of unequal size were observed medial to the right mandible (Figure 4). The short TI inversion recovery (STIR) showed a higher signal in the area of the lesions compared to the surrounding tissues (Figure 5) [3].

A clinical diagnosis of suspected multiple phleboliths within the right mandibular vein was made. There was no swelling, redness, or pain in the right mandible. No aggressive treatment was performed, since the patient preferred to be reviewed at regular intervals by the doctor. To date, the patient has remained asymptomatic.

She was also examined by our otolaryngology department; as she was, asymptomatic, the follow-up protocol was continued.

## 3. Discussion

Venous malformations are the most common among all vascular malformations, with a male-to-female ratio ranging from 1 : 1 to 1 : 2. The size and distribution of venous malformations vary, with 40–50% of all hemangiomas occurring in the head and neck region, particularly in the tongue, buccal mucosa, and lips [3]. The incidence of phleboliths in the head and neck region has been reported to range from 5% to 20% [4].

While the association of hemangiomas with phleboliths has been reported in approximately 5% of all oral surgery cases, internal hemangiomas in the mandible are a much rarer occurrence [5]. The submandibular region is considered the most common site for phleboliths. In the present case, the presence of phleboliths was indicated by areas with high absorption on the magnetic resonance T2-weighted image and STIR image; they were observed on the inner side of the right mandible on computed tomography, outside of the submandibular region.

Phleboliths are characterized by a high degree of calcification and are thought to be caused by the dystrophic calcification of intravenous thrombi [6, 7]. When swelling or hard masses occur in the buccal or submandibular region, it is necessary to distinguish phleboliths from sialoliths in the parotid or submandibular glands [8]. The key to this differentiation is that both painful swelling and salivary colic are observed with sialoliths, whereas painless swelling is associated with phleboliths [8, 9].

While hemangiomas and vascular malformations are often referred to as hemangiomas, the International Society for the Study of Vascular Anomalies (ISSVA) has previously proposed that they should be classified as either vascular tumors or vascular malformations, based on the presence or absence of vascular endothelial cell proliferation [4]. The diagnostic criteria and treatment policy proposed by the ISSVA have recently become internationally standardized. According to the ISSVA classification, an autopsy case is considered a venous malformation. D-dimer levels are elevated in approximately 42% of patients with vascular malformations, due to thrombus formation [4]; the patient in this report had an elevated D-dimer level (36.62 *μ*g/mL), suggesting the presence of a venous malformation.

Inflammatory and cystic masses (e.g., lymphadenitis, cystoid cyst, salivary gland inflammation, calcified epithelioma, and sialolith), benign tumors (e.g., schwannoma, lymphangioma, and pleomorphic adenoma), and malignant lesions (e.g., adenoid cystic carcinoma) have been typically considered in the differential diagnosis of deep-seated hemangiomas in the past.

There was an extensive high-intensity signal on the medial aspect of the right side of the face in the STIR image. Computed tomography clearly differentiated the inferior margin of the right mandible from the submandibular gland, and numerous calcifications were observed. Sialoliths were excluded due to the absence of pain associated with the salivary glands. However, a definitive diagnosis of multiple phleboliths could not be made, as they were not removed, and a biopsy was not performed.

Surgical removal, packing, cryosurgery, tissue sclerotherapy, and radiation therapy [10, 11] have been reported as treatment methods for venous malformations. Nevertheless, the choice of treatment should take into consideration the patient's age, as well as the size, site of origin, and characteristics (including its relationship with surrounding tissues and nutrient vessels) of the tumor. In the present case, after explaining the advantages and disadvantages of surgical treatment to the patient, she elected to undergo observation of the lesion only. To date, no swelling, redness, or pain has been observed in the right side of the mandible.

Taken together, we described a case in which multiple phleboliths were diagnosed in the right mandibular region.

Despite the absence of a cardiovascular event, an elevated D-dimer level was evident. Thus, this case illustrates that even in the absence of symptoms or asymptomatic, facial, or orofacial abnormalities, an elevated D-dimer level may indicate the presence of venous malformations in the head and neck region and the need for further investigations.
